# Loss to follow-up among adults with drug-resistant TB in Papua New Guinea

**DOI:** 10.5588/pha.24.0004

**Published:** 2024-09-01

**Authors:** F. Charles, Y.D. Lin, J. Greig, S. Gurra, R. Morikawa, S.M. Graham, A. Maha

**Affiliations:** ^1^ The Burnet Institute, Port Moresby, Papua New Guinea (PNG);; ^2^ The Burnet Institute, Melbourne, VIC, Australia,; ^3^ Port Moresby General Hospital, Port Moresby, PNG;; ^4^ University of Melbourne Department of Paediatrics, Royal Children’s Hospital, Melbourne, VIC, Australia;; ^5^ National Department of Health, PNG;; ^6^ Gulf Provincial Health Authority.

**Keywords:** treatment outcomes, pre-treatment, bedaquiline, PNG, LTFU, tuberculosis, DR-TB

## Abstract

**SETTING:**

Multidrug-resistant/rifampicin-resistant TB (MDR/RR-TB) is now endemic in the National Capital District (NCD), Papua New Guinea. Loss to follow-up (LTFU) is a challenge.

**OBJECTIVE:**

To evaluate and identify risk factors for LTFU, including pre-treatment LTFU, in adults with MDR/RR-TB at Port Moresby General Hospital (PMGH).

**DESIGN:**

A retrospective analysis of treatment initiation in adults diagnosed with MDR/RR-TB (2018–2022) and outcomes for a cohort treated for MDR/RR-TB (2014-2019). We assessed the factors associated with LTFU using multivariate logistic regression.

**RESULTS:**

Of 95 patients diagnosed with MDR/RR-TB at PMGH from 2018 to 2022, 21 (22%) were lost to follow-up before treatment. Of the 658 adults who initiated treatment for MDR/RR-TB at PMGH from 2014 to 2019, 161 (24%) were lost to follow-up during treatment. A higher proportion of patients on injectable-containing long regimens (110/404, 27%) were lost to follow-up than those on the all-oral regimen containing bedaquiline (13/66, 12%). Treatment loss to follow-up was associated with age (35–54 years age group: aOR 0.49, 95% CI 0.32–0.77; 55–75 years age group: aOR 0.42, 95% CI 0.19–0.90; compared to the 15–34 years age group), residence outside of NCD (aOR 1.79, 95% CI 1.04–3.06), and year of treatment initiation.

**CONCLUSION:**

Pre-treatment LTFU requires programmatic focus. Shorter oral regimens and decentralised services may address the reasons for higher LTFU in younger people and people living outside NCD.

## Abstract (fr)

**CONTEXTE:**

La TB multirésistante/résistante à la rifampicine (MDR-TB/RR-TB, pour l’anglais « *multidrug/rifampicin-resistant TB* ») est maintenant endémique dans le district de la capitale nationale (NCD, pour l’anglais « *National Capital District* »), en Papouasie-Nouvelle-Guinée. La perte de suivi (LTFU, pour l’anglais « *loss to follow-up* ») est un défi.

**OBJECTIF:**

Évaluer et identifier les facteurs de risque de LTFU, y compris le LTFU avant le traitement, chez les adultes atteints de MDR-TB/RR-TB à Port Moresby General Hospital (PMGH).

**CONCEPTION:**

Une analyse rétrospective de l'initiation du traitement chez les adultes diagnostiqués avec une MDR-TB/RR-TB (2018–2022) et des résultats pour une cohorte traitée pour la MDR-TB/RR-TB (2014–2019). Nous avons évalué les facteurs associés au LTFU à l'aide d'une régression logistique multivariée.

**RÉSULTATS:**

Sur les 95 patients diagnostiqués avec une MDR-TB/RR-TB à PMGH de 2018 à 2022, 21 (22%) ont été perdus de vue avant le traitement. Sur les 658 adultes qui ont commencé un traitement pour la MDR-TB/RR-TB à PMGH entre 2014 et 2019, 161 (24%) ont été perdus de vue pendant le traitement. Une proportion plus élevée de patients recevant des régimes longs contenant des injectables (110/404 ; 27%) ont été perdus de vue que ceux recevant un régime entièrement oral contenant de la bédaquiline (13/66 ; 12%). La perte de traitement au suivi était associée à l'âge (groupe d'âge de 35 à 54 ans : aOR 0,49 ; IC à 95% 0,32 à 0,77 ; groupe d'âge de 55 à 75 ans : aOR 0,42 ; IC à 95% 0,19 à 0,90 ; par rapport au groupe d'âge de 15 à 34 ans), à la résidence en dehors des NCD (aOR 1,79 ; IC à 95% 1,04 à 3,06) et à quelques années de début de traitement.

**CONCLUSION:**

Le LTFU avant le traitement nécessite une orientation programmatique. Des régimes oraux plus courts et des services décentralisés peuvent s'attaquer aux raisons de l'augmentation du LTFU chez les jeunes et les personnes vivant en dehors des NCD.

TB remains a public health concern, with a global incidence of 134/100,000 for drug-susceptible TB (DS-TB) and 5.7/100,000 for multidrug-resistant/rifampicin-resistant TB (MDR/RR-TB).^1^ It is a leading cause of death, with 50% mortality if untreated. Papua New Guinea (PNG) is listed by the WHO among the 30 high-burden countries for TB and MDR/RR-TB, with a TB incidence in 2022 of 432/100,000 and MDR/RR-TB incidence of 22/100,000, population.^1^ There is a heightened public health risk of MDR/RR-TB in settings where there are suboptimal outcomes, such as high rates of loss to follow-up (LTFU).^2–4^

The prevalence of drug-resistant TB (DR-TB) varies among different groups in PNG. A limited population survey in 2012–2014 reported that the overall prevalence of MDR/RR-TB was 23% in retreatment cases and 4% in new cases, but that specific populations accounted for a disproportionately high number of cases.^5^ A high prevalence of MDR/RR-TB has been noted in new TB cases in the South Fly District of Western Province, with evidence that community transmission of DR-TB is common in that population.^6,7^ A review of laboratory data since the implementation of GeneXpert (Cepheid, Sunnyvale, CA) in PNG reported that the majority of 770 samples with drug resistance confirmed in the reference laboratory were from either Western province (34.8%) or the National Capital District (NCD) (46.5%), which includes Port Moresby, the capital of PNG.^8^ However, this partly reflects underutilisation of molecular diagnostics and lack of capacity to diagnose resistance in other parts of PNG.

Treatment outcomes for all TB, including MDR/RR-TB, are a major programmatic challenge in PNG.^9^ The treatment success rate for 229 people with MDR/RR-TB who initiated treatment in 2020 was reported as only 72% compared with the global target of 90%.^1^ Of particular note is the persistently high rate of treatment LTFU for DS- and MDR-TB.^9^ There is also concern regarding pre-treatment LTFU, although PNG data are very limited.^10^ Pre-treatment LTFU is not routinely reported by programmes, but may be an important reason for patient losses in the cascade of care. A systematic review of 23 studies from 14 countries reported that 4% to 38% of people with TB experienced pre-treatment LTFU.^11^ LTFU for TB, including pre-treatment LTFU for MDR/RR-TB are likely to be important drivers of the emergence and community transmission of DR-TB. Understanding risk factors associated with LTFU would help determine locally relevant interventions, but this has not been evaluated in PNG.

We aimed to describe the characteristics and treatment outcomes for MDR/RR-TB, including pre-treatment LTFU in patients presenting for care at PNG’s largest TB and MDR/RR-TB treatment facility and to determine factors associated with LTFU.

## METHODS

We conducted a retrospective analysis of bacteriologically confirmed MDR/RR-TB cases at the TB clinic at Port Moresby General Hospital (PMGH) to determine: pre-treatment initiation LTFU in presumptive TB cases diagnosed with MDR/RR-TB at PMGH from 2018 to 2022; and treatment LTFU for people commencing treatment for MDR/RR-TB from 2014 to 2019.

### Study setting and population

PNG is a middle-income country located in the Pacific Region, with 22 provinces over four regions and a population of approximately 12 million.^12^ Approximately three-quarters of the population are aged less than 35 years and 80% of the population reside in rural areas, many of which are geographically remote.^12,13^ Deteriorating government services in rural PNG has pushed increased rural-urban migration, leading to an increasing disadvantaged population in urban centres, most prominently in the NCD of PNG.^14^ The NCD has three districts with a combined population of almost 500,000.^12^ Most of this population is not formally employed and lives in overcrowded squatter settlements with limited access to the already overburdened public healthcare facilities.

PMGH is the tertiary referral hospital of the country, but its location also makes it the primary healthcare provider to the disadvantaged population in the NCD and neighbouring Central and Gulf provinces. PMGH serves as one of the 10 TB Basic Management Units (BMUs). In 2012, PMGH was the first site to provide care for people with MDR/RR-TB from NCD, Central and Gulf provinces. From 2018, two additional programmatic management of drug-resistant TB (PMDT) sites were established to address the increasing DR-TB burden. Three urban PMDT sites are each responsible for one district of the NCD, while PMGH continues to provide PMDT services for DR-TB cases from neighbouring provinces.

The Xpert MTB/RIF assay was introduced in 2012 to improve the diagnostic capacity of TB.8 Initially, it was only used for groups considered to be at risk of MDR/RR-TB, including retreatment cases, treatment failure during treatment, MDR/RR-TB contacts and people living with HIV. In 2018, Xpert MTB/RIF became the primary diagnostic tool for people with presumptive TB.^15^

Treatment was initiated at PMGH, and clinical monitoring at monthly follow-up was provided at the nearest established PMDT site. Directly observed therapy and treatment adherence are monitored daily at the nearest BMU. PMGH functions as a PMDT site as well as a BMU for those who live locally. Since 2018, BMU activities have been strengthened with community treatment supporters; patient enabling programmes (food vouchers and bus fares), and patient education and counselling. These activities are mostly dependent on partner organisations with the associated risk of discontinuity or interruption.

### Study population and definitions

The study population consisted of people aged 15 years and older with presumptive TB and bacteriologically confirmed MDR/RR-TB who were registered in the presumptive TB and PMDT registers, respectively. Pre-treatment LTFU was defined as a person with bacteriologically confirmed MDR/RR-TB recorded in the presumptive TB register but not registered for treatment in any PMDT register. For those who initiated treatment, we used the standard definitions for treatment outcomes from the PNG PMDT guidelines. Treatment LTFU was defined as treatment interruption for ≥2 months in persons with bacteriologically confirmed MDR/RR-TB.^16^

### Data collection and analysis

Clinical and demographic characteristics of people with presumptive TB and MDR/RR-TB detected using Xpert MTB/RIF were extracted from the PMGH presumptive TB registers from 2018 to 2022. The MDR/RR-TB cases identified in the presumptive TB register were cross-checked against the PMDT registers, which included PMGH and other PMDT sites. All MDR/RR-TB cases who were not registered in any PMDT register were identified as pre-treatment LTFU.

Clinical and demographic characteristics and the treatment outcomes of people commenced on treatment at PMGH were extracted from the PMDT registers from 2014 to 2019. People who transferred out (*n =* 22) were excluded. Information missing in the register was collected from treatment cards and admission charts where possible.

Data collected from the registers were entered into a password-protected MS 365 Excel (Microsoft, Redmond, WA, USA) workbook with data validation restrictions. Variables included demographic characteristics, diagnostics used and results, TB disease site and category, key risk factors, treatment regimen and treatment outcomes. For the primary analysis, the outcomes were classified as LTFU or not LTFU (all other outcomes). Categorical variables were described by frequency (percentage), and continuous variables by median (interquartile range [IQR]). Differences in categorical variables were tested using the χ^2^ test; differences between continuous variables were tested using the non-parametric Wilcoxon rank-sum test. Logistic regression analysis was used to explore demographic and clinical factors associated with LTFU, with confounders assessed through univariate analysis and then multivariate logistic regression with backward selection retaining only variables significantly associated with the outcome. Associations are reported as odds ratios (ORs) with 95% confidence intervals (CIs) and *P* < 0.05 was considered statistically significant. Data were analysed using STATA v.17 (StataCorp, College Station, TX, USA).

### Ethics approval

Ethics approval was obtained from PNG Medical Research Advisory Council (MRAC).

## RESULTS

There were 5,469 adults registered in the presumptive TB register who also underwent an Xpert MTB/RIF test at PMGH from 2018 to 2022 (Table 1). Of these, 1,027 (19%) had MTB detected by Xpert, and 95 (2%) were diagnosed with MDR/RR-TB. Of these, 21 (22%) were lost to follow-up before being initiated on treatment, ranging from 16% to 38% by year (Table 1). More females than males were diagnosed, but the proportion with pre-treatment LTFU was similar by sex (Table 2). The 15–34 years age group had the highest proportion of people with MDR/RR-TB (67%) and the same proportion were pre-treatment LTFU (67%). Only one person was diagnosed with extrapulmonary MDR/RR-TB. None of the factors recorded in the presumptive TB register were significantly associated with pre-treatment LTFU when assessed using logistic regression; however, there was limited power to detect associations. Of the people who started treatment, 92% (68/74) were treated at PMGH, with the remainder treated at other facilities. Among those commencing MDR/RR-TB treatment at PMGH in 2018, 2019 and 2020, 23% (10/44) were lost to follow-up during treatment and 7% (3/44) had an outcome recorded as not evaluated with one transfer out.

**TABLE 1.** Presumptive TB cases with rifampicin resistance after treatment initiation.

Category	Total	2018	2019	2020	2021	2022
	*n* (%)	*n* (%)	*n* (%)	*n* (%)	*n* (%)	*n* (%)
Presumptive TB case with Xpert test, *n*	5,469	874	1,563	925	766	1,341
MTB detected on Xpert (% of presumptive TB)	1,027 (19)	116 (13)	269 (17)	237 (26)	170 (22)	235 (18)
RR-TB detected on Xpert (% of presumptive TB)	95 (2)	22 (3)	32 (2)	8 (1)	15 (2)	18 (1)
RR-TB registered in the PMDT (% of RR-TB detected)	74 (78)	16 (73)	27 (84)	5 (63)	12 (80)	14 (78)
Pre-treatment LTFU (% of RR-TB detected)	21 (22)	6 (27)	5 (16)	3 (38)	3 (20)	4 (22)

MTB = *M*. *tuberculosis*; RR-TB = rifampicin-resistant TB; PMDT = programmatic management of drug-resistant TB; LTFU = loss to follow-up.

**TABLE 2.** Characteristics by treatment status (registered vs. pre-treatment LTFU) of people diagnosed with rifampicin-resistant TB at PMGH, Port Moresby, PNG, 2018–2022.*

	Total	Registered	Pre-treatment LTFU	*P*-value^†^
	(*n* = 95)	(*n* = 74)	(*n* = 21)	
	*n* (%)	*n* (%)	*n* (%)	
Sex				
Male	40 (42)	29 (39)	11 (52)	0.51
Female	54 (57)	44 (59)	10 (48)	
Unknown	1 (1)	1 (1)	0 (0)	
Age, years, median [IQR]^‡^	28 [24–38]	28 [24–37]	29 [21–40]	0.94
15–34	63 (67)	49 (67)	14 (67)	0.73
35–54	23 (24)	17 (23)	6 (29)	
55–75	8 (9)	7 (10)	1 (5)	
Place of residence				
Port Moresby South	23 (24)	17 (23)	6 (29)	0.83
Port Moresby East	38 (40)	32 (43)	6 (29)	
Port Moresby West	11 (12)	8 (11)	3 (14)	
Outside NCD	19 (20)	14 (19)	5 (24)	
Unknown	4 (4)	3 (4)	1 (5)	
Site of TB				
Pulmonary	92 (97)	73 (99)	19 (90)	0.024
Extrapulmonary	1 (1)	1 (1)	0 (0)	
Unknown	2 (2)	0 (0)	2 (10)	
HIV status: HIV infected	10 (11%)	10 (14)	0 (0)	

* Cells show count and proportion within the column, except age.

† χ^2^ test for counts and Wilcoxon rank-sum tests for continuous variables.

‡ Age unknown *n =* 1 (registered for treatment).

LTFU = loss to follow-up; PMGH = Port Moresby General Hospital; PNG = Papua New Guinea; NCD = National Capital District.

A total of 658 persons were registered and treated for bacteriologically confirmed MDR/RR-TB at PMGH from 2014 to 2019 (Table 3). Most patients were referred to PMGH by other facilities after diagnosis, with only 12% in 2018-2019 in the PMGH pre-treatment (presumptive) register. The overall treatment success (outcome cured or treatment completed) rate was 50%, with 24% of patients lost to follow-up during MDR/RR-TB treatment; 14% of treated patients died. The proportion of LTFU decreased from 38% for treatments commenced in 2014 to 18% in 2019, even as the annual MDR/RR-TB treatment caseload increased from 69 to 194 (Figure). LTFU occurred after a median of 6 months (IQR 2–12). There was a notable rise in treatment completion in 2019, but overall treatment success remained relatively constant over time because the proportion confirmed as cured decreased (Figure).

**TABLE 3.** Factors associated with LTFU treatment outcome for people treated for MDR/RR-TB at PMGH, Port Moresby, PNG, for treatments starting between 2014 and 2019.

	Total assessed	LTFU outcome	Not LTFU*	OR (95% CI)	*P-*value	aOR (95% CI)	*P-*value
	(*n* = 658)	(*n* = 161)	(*n* = 497)				
	*n* (%)	*n* (%)	*n* (%)				
Sex							
Male	284 (43)	72 (45)	212 (43)	1.00			
Female	374 (57)	89 (55)	285 (57)	0.92 (0.64–1.32)	0.65		
Age, years, median [IQR]	30 [23–40]	28 [22–36]	31 [24–41]	0.98 (0.96–0.99)	0.008		
Age groups, years							
15–34	406 (62)	116 (72)	290 (58)	0.57 (0.37–0.87)		1.00	
35–54	194 (29)	36 (22)	158 (32)	0.46 (0.22–0.97)	0.009	0.49 (0.32–0.77)	0.002
55–75	58 (9)	9 (6)	49 (10)	1.00	0.040	0.42 (0.19–0.90)	0.025
Place of residence^†^				0.87 (0.55–1.37)			
Port Moresby South	184 (28)	44 (27)	140 (28)	1.03 (0.58–1.83)		1.00	
Port Moresby East	256 (39)	55 (34)	201 (40)	1.55 (0.93–2.59)	0.55	0.98 (0.61–1.56)	0.92
Port Moresby West	98 (15)	24 (15)	74 (15)	1.00	0.91	0.92 (0.50–1.70)	0.80
Port Moresby NCD	116 (18)	38 (24)	78 (16)	1.20 (0.71–2.04)	0.095	1.79 (1.04–3.06)	0.034
Site of TB disease							
PTB	543 (82)	132 (82)	411 (83)	1.00			
EPTB	79 (12)	22 (14)	57 (11)	0.92 (0.64–1.32)	0.50		
PTB+EPTB	36 (6)	7 (4)	29 (6)	0.98 (0.96–0.99)	0.51		
Type of regimen							
Long regimen injectable	404 (61)	110 (68)	294 (59)	1.00			
Short regimen injectable	162 (25)	35 (22)	127 (26)	0.74 (0.48–1.14)	0.17		
All-oral long-term regimen	26 (4)	3 (2)	23 (4)	0.35 (0.10–1.18)	0.091		
Individualised regimen	66 (10)	13 (8)	53 (11)	0.66 (0.34–1.25)	0.20		
Culture result							
MTB growth	428 (65)	110 (68)	318 (64)	1.00			
No growth	55 (8)	10 (6)	45 (9)	0.64 (0.31–1.32)	0.23		
Unknown	175 (27)	41 (26)	134 (27)	0.88 (0.59–1.33)	0.56		
Year treatment was initiated^‡^							
2013	5 (1)	2 (1)	3 (1)	3.14 (0.50–19.5)	0.22	3.85	0.16
2014	69 (10)	26 (16)	43 (9)	2.85 (1.54–5.25)	0.0008	3.27	0.0003
2015	70 (11)	22 (14)	48 (10)	2.16 (1.15–4.03)	0.016	2.22	0.017
2016	113 (17)	21 (13)	92 (18)	1.07 (0.59–1.96)	0.82	1.12	0.72
2017	94 (14)	24 (15)	70 (14)	1.61 (0.89–2.92)	0.11	1.62	0.12
2018	112 (17)	32 (10)	80 (16)	1.88 (1.08–3.27)	0.025	2.01	0.016
2019	194 (29)	34 (21)	160 (32)	1.00		1.00	
Time to treatment initiation after the Xpert test, days^§^							
0–30	414 (71)	89 (64)	325 (73)	1.00			
>30	171 (29)	51 (36)	120 (27)	1.55 (1.04–2.32)	0.032		

* All other outcomes, including not evaluated.

† Excludes ‘Unknown’ (*n* = 4).

‡ Excludes the year 2012 (*n =*1).

§ Number of days from an Xpert MTB/RIF-positive result and the date of treatment initiation.

LTFU = loss to follow-up; MDR/RR-TB = multidrug-resistant/rifampicin-resistant TB; PMGH = Port Moresby General Hospital; PNG = Papua New Guinea; OR = odds ratio; CI = confidence interval; aOR = adjusted OR; IQR = interquartile range; NCD = National Capital District; PTB = pulmonary TB; EPTB = extrapulmonary TB; MTB = *M. tuberculosis*.

**FIGURE.**
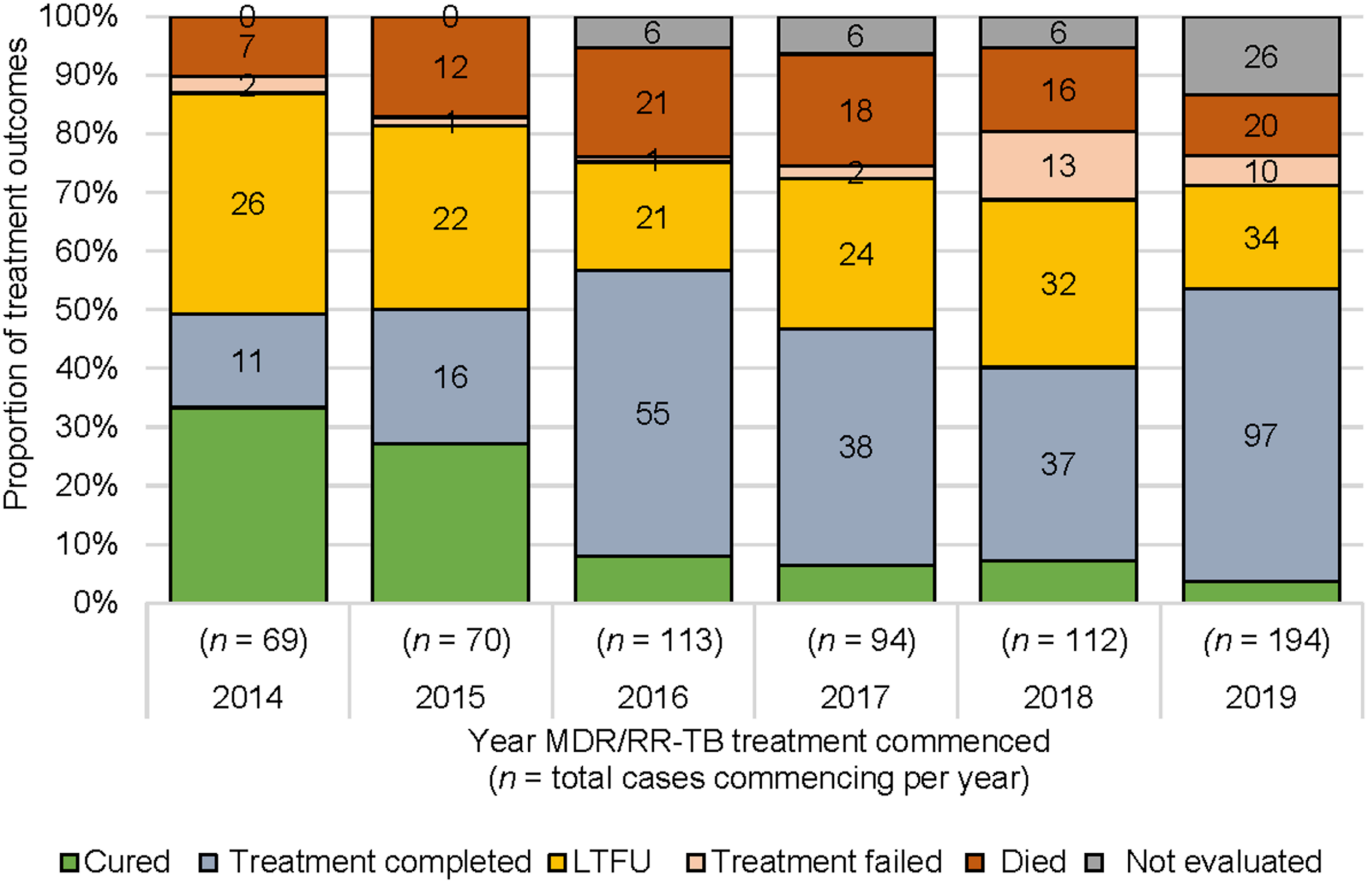
Treatment outcomes of adults registered in PMGH for MDR/RR-TB treatments from 2014 to 2019. MDR/RR-TB = multidrug/rifampicin-resistant TB; LTFU = loss to follow-up; PMGH = Port Moresby General Hospital.

The majority (62%) of people commencing treatment were aged 15–34 years, but a higher proportion of LTFU (72%) were in this age group (Table 3). Of the people treated, 18% were listed as living outside NCD, and they constituted 24% of those lost to follow-up. Most (61%) people were treated with an injectable-containing longer regimen, which represented 68% of LTFU. The proportion of patients who received the all-oral regimen containing bedaquiline (BDQ) was low, as BDQ was only introduced in 2019; 4% of the cohort and 2% of those lost to follow-up.

Table 3 shows the results of the analysis of factors associated with LTFU. In multivariate analysis, treatment LTFU was associated with the younger age category and living outside NCD. Those aged 35–54 years and 55–75 years had lower odds of LTFU compared with those aged 15–34 years (aOR 0.49, 95% CI 0.32–0.77 and aOR 0.42, 95% CI 0.19–0.90 respectively). Living outside NCD was associated with increased odds of treatment LTFU (aOR 1.79, 95%CI 1.04–3.06). We also identified a significant association between LTFU and treatments commenced in 2014, 2015 and 2018 (respectively aOR 3.27, 95% CI 1.71–6.22); aOR 2.22, 95% CI 1.15–4.27; aOR 2.01, 95% CI 1.14–3.54). In 2014 and 2015, the only standardised regimen in use was the injectable-containing long regimen, with individualised regimens given to only 6% in 2014 and 13% in 2015.

## DISCUSSION

LTFU is a major challenge in adults with MDR/RR-TB who present for care at PNG’s largest TB clinic. The high rate of pre-treatment LTFU is an important observation, as pre-treatment LTFU is not reported to the NTP. The NTP of PNG reported to the WHO that there were 656 laboratory-confirmed MDR/RR-TB cases detected in PNG in 2022 and that 567 people initiated treatment for MDR/RR-TB in the same year.^1^ While it is not possible to determine national pre-treatment LTFU rates from these reported data, our finding of 22% is similar to that reported from another province of PNG; 21% of 58 sputum smear-positive cases recorded in the laboratory register did not initiate treatment in 2014–2015.^10^ This is a major concern that needs to be addressed to improve health outcomes for people with TB and to reduce ongoing transmission in the community.

We did not identify potentially modifiable factors associated with pre-treatment LTFU, but the cohort for analysis was not large (*n =* 95) because only a small proportion of those treated at PMGH were diagnosed there. It would be valuable to similarly assess pre-treatment LTFU in the surrounding facilities in NCD that diagnose and refer patients to PMGH for treatment, as well as elsewhere in PNG. Factors that are not recorded in the registers may be relevant to pre-treatment LTFU and may warrant exploration, such as accessibility, delay in diagnostic turn-around-time, socio-economic and psychological issues.^17,18^ Provision of information to locate an individual, such as a mobile phone number or address, is also potentially useful.

MDR/RR-TB treatment outcomes had a reduced cure rate after 2015, but the treatment completion rate increased such that overall success was relatively stable; this change reflects the unavailability of routine culture. The treatment success rate has remained low (average 50%) compared with the NTP target of 90%. The main unsuccessful outcome was LTFU, which at 22% is much higher than the NTP target of <5%, and LTFU was significantly higher in people aged 15–34 years. This young population is not only vulnerable to TB disease sequelae, but untreated patients are also likely to contribute to the community transmission of TB infection and disease.^19,20^ Treatment LTFU was also associated with living outside NCD, which may reflect distance- and living condition-related challenges to health equity (accessibility, socio-economic and psychological factors). Decentralisation of services for TB, including the introduction of rapid molecular diagnostics to the primary care level, can improve treatment access and coverage while maintaining treatment outcomes.^21,22^ The recent introduction of all-oral and shorter regimens for MDR/RR-TB provides the opportunity and potential for a similar model of care.

Treatment regimens were not retained in the logistic regression model, but changes in risks for treatment LTFU over time were at least partly related to the regimen used, and this changed over the years assessed. A high proportion of people on an injectable-containing longer regimen had treatment LTFU compared to an all-oral regimen of similar duration containing bedaquiline. Regimens used in 2014 and 2015, years with significantly higher LTFU, were mostly injectable-containing longer regimens. LTFU may be attributable to less desirable aspects of the injectable-containing regimen, including: longer treatment duration, high pill burden, drugs with greater toxicity, and requiring painful intramuscular injections for up to 8 months. Access to services for daily injections may also be a major challenge, especially for those who live remotely at a distance from clinics.18 The significant association of high treatment LTFU in 2018, which could include treatments ending in 2020, may reflect programmatic changes and challenges including: treatment regimens, diagnostic algorithms, health services disruption due to national elections, and the impact of COVID-19 on health services.

Having a relatively large cohort of MDR/RR-TB treatment data is a strength of this study. Study limitations included pre-treatment data available only from 2018 onwards and only for PMGH, imperfect data held in paper registers and potential misrepresentation of current patient addresses. Other variables like socio-economic status that may contribute to LTFU before treatment and during treatment are not collected programmatically. While pre-treatment data were not available from 2014 to fully overlap with the treatment cohorts assessed, the treatment initiation status for all presumptive TB cases with rifampicin resistance was ascertained over five consecutive years from 2018 to 2022. Data from PMGH may not be representative of other facilities in PNG but represent an important cohort because many MDR/RR-TB cases are treated at PMGH. Molecular analysis of MDR-TB clusters in PNG showed that the majority involved at least one strain from NCD, which highlights the fact that NCD is a site of amplification of MDR transmission to other provinces.^23^ Thus, pre-retreatment and treatment LTFU in NCD is a challenge to TB elimination across all of PNG.

In 2021, the WHO aligned treatment outcomes for DR-TB with those for DS-TB and lost to follow-up now includes ‘a patient who did not start treatment’.^24^ In PNG, NTP reporting templates should be altered to include reporting of pre-treatment LTFU from the facility level. This would provide critical nationwide data for monitoring and evaluating this important outcome. A digital case-based TB register is being implemented in the PNG electronic National Health Information System, which offers the potential for improved cohort monitoring, but could not currently facilitate early identification of those missing follow-ups. Timely interventions to improve the initiation and completion of treatment require monitoring that identifies people needing more support early so that they do not miss care long enough to be designated as lost to follow-up. To determine the factors associated with LTFU, it would also be valuable to conduct prospective studies in a range of settings. This should include qualitative exploration of a range of factors such as socio-economic status, migration, stigma and adverse effects that could inform targeted support measures (such as peer education and counselling, social protection) to improve treatment commencement and completion, particularly in the younger population.

In conclusion, the high rates of pre-treatment and treatment LTFU in people diagnosed with MDR/RR-TB at PNG’s largest treatment facility are a major concern that requires prospective evaluation of risk factors and similar analysis in a range of settings in PNG. WHO-recommended rapid diagnostics and all-oral treatment regimens containing Bedaquiline could facilitate access to treatment, but strengthening of services is urgently required to reduce LTFU and improve treatment outcomes.
